# Population Density, Water Supply, and the Risk of Dengue Fever in Vietnam: Cohort Study and Spatial Analysis

**DOI:** 10.1371/journal.pmed.1001082

**Published:** 2011-08-30

**Authors:** Wolf-Peter Schmidt, Motoi Suzuki, Vu Dinh Thiem, Richard G. White, Ataru Tsuzuki, Lay-Myint Yoshida, Hideki Yanai, Ubydul Haque, Le Huu Tho, Dang Duc Anh, Koya Ariyoshi

**Affiliations:** 1 Department of Clinical Medicine, Institute of Tropical Medicine, Nagasaki University, Nagasaki, Japan; 2 National Institute of Hygiene and Epidemiology, Hanoi, Vietnam; 3 Department of Infectious Disease Epidemiology, London School of Hygiene and Tropical Medicine, London, United Kingdom; 4 Department of Vector Ecology and Environment, Institute of Tropical Medicine, Nagasaki University, Nagasaki, Japan; 5 Department of Mathematical Sciences and Technology, Norwegian University of Life Sciences, Aas, Norway; 6 Khanh Hoa Health Service, Nha Trang, Khanh Hoa, Vietnam; 7 Global COE Program, Nagasaki University, Nagasaki, Japan; Oxford University Clinical Research Unit, Vietnam

## Abstract

**Background:**

*Aedes aegypti*, the major vector of dengue viruses, often breeds in water storage containers used by households without tap water supply, and occurs in high numbers even in dense urban areas. We analysed the interaction between human population density and lack of tap water as a cause of dengue fever outbreaks with the aim of identifying geographic areas at highest risk.

**Methods and Findings:**

We conducted an individual-level cohort study in a population of 75,000 geo-referenced households in Vietnam over the course of two epidemics, on the basis of dengue hospital admissions (*n* = 3,013). We applied space-time scan statistics and mathematical models to confirm the findings. We identified a surprisingly narrow range of critical human population densities between around 3,000 to 7,000 people/km^2^ prone to dengue outbreaks. In the study area, this population density was typical of villages and some peri-urban areas. Scan statistics showed that areas with a high population density or adequate water supply did not experience severe outbreaks. The risk of dengue was higher in rural than in urban areas, largely explained by lack of piped water supply, and in human population densities more often falling within the critical range. Mathematical modeling suggests that simple assumptions regarding area-level vector/host ratios may explain the occurrence of outbreaks.

**Conclusions:**

Rural areas may contribute at least as much to the dissemination of dengue fever as cities. Improving water supply and vector control in areas with a human population density critical for dengue transmission could increase the efficiency of control efforts.

*Please see later in the article for the Editors' Summary*

## Abstract 2

Results from 75,000 geo-referenced households in Vietnam during two dengue epidemics reveal that human population densities typical of villages are most prone to dengue outbreaks; rural areas may contribute as much to dissemination of dengue fever as do cities.

## Editors' Summary

**Background:**

Dengue fever is a viral infection common in tropical and subtropical regions that is characterized by sudden high fever, severe headache, muscle and joint pains, and a rash. The virus is transmitted by the bite of female *Aedes aegypti* mosquitoes. Although dengue is not usually fatal, infection rates can be as high as 90% among those who have not been previously exposed to the virus, and in a small proportion of cases the disease can develop into dengue hemorrhagic fever, which is life threatening. It is estimated that 500,000 people are hospitalized every year with dengue hemorrhagic fever. Incidence of dengue fever is increasing, and two-fifths of the world's population, approximately 2.5 billion people, are now at risk from the disease in over 100 endemic countries.

**Why Was This Study Done?:**

There is no specific treatment for dengue fever, other than managing symptoms and ensuring hydration, and no vaccine available. The best way to counter the spread of dengue fever is to target the mosquito vector, with one of the more effective methods being the disruption of mosquito habitats, in particular eliminating standing water such as in unused tires, open water storage containers, or even flower vases, where mosquitoes lay their eggs and larvae develop. Because the geographic range of the mosquitoes that transmit dengue has increased, there has been a rapid rise in global dengue epidemics over the last 30 years with Southeast Asia and the Western Pacific being most severely affected. In this study researchers aimed to define areas in Vietnam that were most at risk of dengue fever by looking at population density and water supply.

**What Did the Researchers Do and Find?:**

The researchers studied a population in Kanh-Hoa Province in south-central Vietnam (∼350,000 people) that was affected by two dengue epidemics between January 2005 and June 2008. They included all patients admitted to two public hospitals that could be linked to census data from 2006 (3,013 patients). These data enabled the researchers to calculate both the population density and the proportion of households with access to tap water within 100 meters of each patient's household.

The researchers found that low population densities, typical of rural villages (around 110 people residing within a 100-meter radius), had the highest rate of dengue fever. They also found that in those neighborhoods where less than 20% of households had tap water there was a peak in dengue fever rates at a population density of 190 people residing within 100 meters. On an individual household level they found that absence of tap water was associated with an increased risk of dengue fever.

In the absence of data on larvae and mosquito abundance the researchers used a mathematical model to show that when mosquito numbers were limited the highest risk of dengue occurred at very low population densities. However, if mosquito numbers were limited only at high human population densities, dengue fever risk peaked at low-to-moderate human population densities. The model suggests that the provision of tap water changes the risk of dengue because mosquito numbers are limited.

**What Do These Findings Mean?:**

People living in low-to-moderate population densities, such as rural villages, without access to tap water have the highest risk of contracting dengue fever. The use of water storage vessels provides breeding sites for mosquitoes and leads to a high mosquito-to-human ratio and an increased individual dengue risk. In more populated urban areas with tap water, mosquito breeding sites are limited so the relative risk of dengue for an individual is less because the mosquito-to-human ratio is smaller. Populated areas still contribute substantially to dengue epidemics, however, because the absolute number of people who can contract dengue is high.

The authors point out some limitations in their study, such as only looking at the most severe cases of dengue in patients who were admitted to hospital and assuming that all taps were functional.

**Additional Information:**

Please access these Web sites via the online version of this summary at http://dx.doi.org/10.1371/journal.pmed.1001082.

- WHO provides information on dengue fever including a dengue fact sheet
- The CDC provides information on the Aedes aegypti mosquito and a global health map that reports areas at risk of dengue

## Introduction

Dengue viruses cause an estimated 50 million infections annually among approximately 2.5 billion people at risk [1]. The main mosquito vector (*Ae. aegypti*) typically breeds well in human-made container habitats such as water storage jars in and around human settlements including those in dense urban areas [2],[3]. This breeding behavior stands in contrast to most *Anopheles* species (the vector for malaria), which usually avoid urban ecosystems, leading to a low malaria risk in cities [4]. Because *Ae. aegypti* predominantly bites during daylight hours, insecticide-treated bednets may not be very effective in controlling dengue. In the absence of a vaccine, dengue control focuses on reducing vector abundance through insecticides, biological control of larvae, or measures to reduce breeding sites [5]–[7].

Previous studies, including mathematical models, have investigated the effect of climate change [8], demographic transition [9] and urban structure [2],[10] on dengue transmission. High human population density and inadequate water supply (requiring water storage) are regarded as major contributors to dengue epidemics [11],[12], but data in support of these assumptions are scarce. Rural areas with a low population density also experience severe epidemics [13],[14]. The role of human population density and socio-economic factors (especially water supply infrastructure) as risk factors for dengue fever is poorly understood. Population-based studies have provided important insights into the epidemiology of dengue fever, but often have been small, generally relied on cross-sectional seroprevalence data (rather than incidence) and have not quantified human population density as a risk factor [15]–[18].

We analysed the effect of population density and lack of tap water supply on the risk of dengue fever by linking detailed household data from a large census area in Vietnam with hospital admission records.

## Methods

### Study Area and Population

The study area comprised 33 rural and urban communes in the districts Nha Trang and Ninh Hoa, both in Kanh Hoa Province in south-central coastal Vietnam. Communes consisting predominantly of nonresidential, commercial, or holiday resort areas were excluded. In mid-2006 a census was carried out in all existing households in the 33 communes as part of the Khan Hoa Health Project [19].

Khan Hoa Health Project is an ongoing research collaboration between the National Institute of Hygiene and Epidemiology, Hanoi, Vietnam, and Nagasaki University, funded by the Program of Founding Research Centres for Emerging and Re-emerging Infectious Diseases of the Japanese government [19]. The census was led by local health authorities. Participation was near complete. The census included questionnaires covering household demographics, socio-economic factors (education, household appliances, water supply, housing), occupation, and animal ownership. All households were geo-referenced using GPS receivers. In more densely populated areas, households sharing the same small building were geo-referenced as a single location.

Government regulation specifies that two public hospitals, Khanh Hoa General Hospital and Ninh Hoa District Hospital, treat all inpatients in the area. Patient data are continuously entered into a database, allowing linkage between individual patients and census data [19]. Khan Hoa Health Project was approved by the Institutional Review Board at the National Institute of Hygiene and Epidemiology, Hanoi, and the Ethics Committee of the Institute of Tropical Medicine at Nagasaki University. Anonymised data were used for this analysis.

### Exposure Measures

For every household included in the census we calculated the proportion of households without access to tap water within a 100-m radius using ArcGIS 9.2 (ESRI Corporation). Human population density was calculated as the number of people residing within a 100-m radius of the household. A 100-m radius was chosen a priori as a plausible flight range of *Ae. aegypti* [2],[20],[21]. We used the highest level of education of any household member as a household level variable. Household economic status was modeled as a wealth index on the basis of durable assets used previously [22].

### Outcome Measure

**Figure 1:**
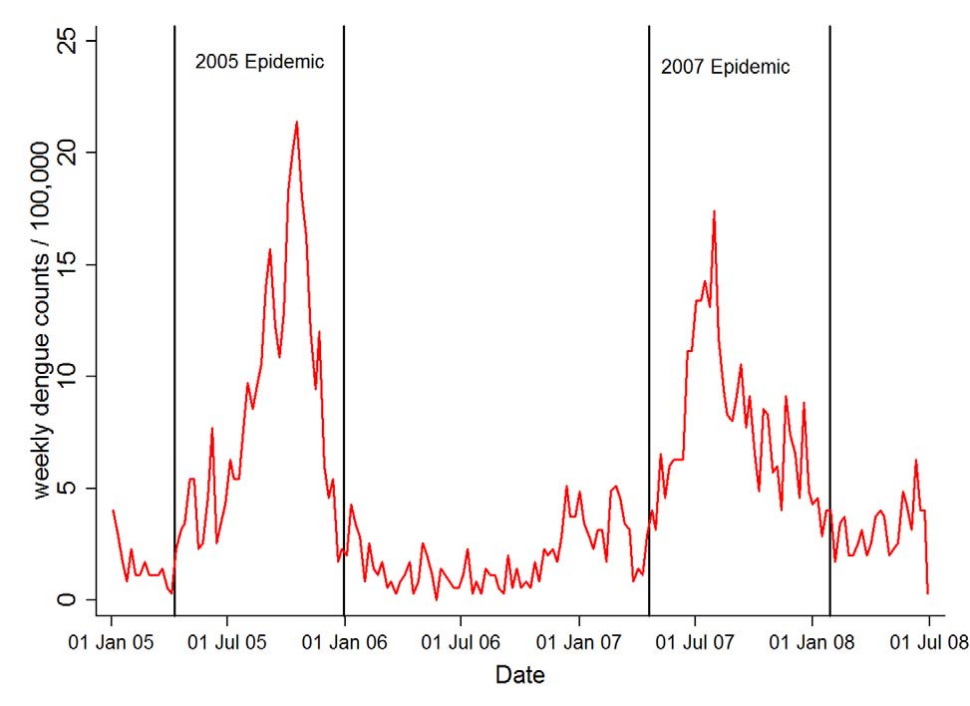
Weekly hospital admission for dengue fever during study period. Vertical lines indicate the approximate beginning and end of the two major epidemics.

Two distinct dengue fever epidemics occurred during the study period between January 2005 and June 2008 (Figure 1). We included dengue cases of all ages from the study area admitted to the two hospitals between January 2005 and June 2008 if they could be linked to the census (70.3% of all admitted dengue cases). Diagnosis of dengue was made following the same standard procedures at both hospitals. Initial clinical diagnosis was based on standard World Health Organization (WHO) criteria [23]. Cases were classified as classic dengue fever or dengue haemorrhagic fever according to symptoms. Every suspected case was confirmed by a single rapid test (SD Bioline Dengue IgG/IgM, SD Bio Standard Diagnostics). If the test was negative despite clinical evidence suggesting dengue, an antigen ELISA test was performed (Platelia(TM) Dengue Ns1 AG, Bio-Rad). Diagnosis of dengue was restricted to patients positive for either test.

### Statistical Analysis

Admission rate was modeled as an open cohort using Poisson regression since children were born into the cohort between January 2005 and mid 2006 (the time of the census). There was no evidence of over-dispersion due to repeat admissions. We considered the whole population at risk throughout the study period between January 2005 and June 2008. Human population density and neighborhood tap water coverage were modeled first as categorical variables and then as restricted cubic splines. Confidence intervals were adjusted for clustering of households with the same geographic coordinates using robust standard errors. These calculations were done in STATA 10 (Statacorp).

We used space-time scan statistics (SaTScan, www.satscan.org) to identify clusters of dengue in space and time [24]. This statistics is an extension of conventional Poisson regression and applies a cylindrical window of increasing diameter to each location with time being represented by the height of the cylinder. We set a radius of 2 km as the upper limit for the scanning window. For computational reasons we averaged the locations of households within 200-m grid cells. To explore the evolving epidemics we divided each a priori into three parts of equal duration (early, middle, late stage). The likelihood ratio tests used in the scan statistics were adjusted for distance to the nearest hospital, wealth, and education, averaged at the 200-m grid level.

### Mathematical Model

Since mosquitoes feed on humans, and since breeding sites are created or destroyed by human activities, it is likely that mosquito density varies with human population density. In this study, we had no field data on mosquito or larval density and were therefore unable to calculate the vector/host ratio directly. In order to explore the association between vector abundance and human population density, and its effect on dengue fever risk, we applied a simple mathematical model on the basis of the classic Ross-MacDonald model [25], which can be formulated as follows [26]:
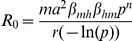
where

*m* = ratio of the number of mosquitoes to number of humans
*a* = number of human bloodmeals per mosquito per day
β_mh_ = probability of transmission mosquito to human
β_hm_ = probability of transmission human to mosquito
*p* = mosquito daily survival probability
*n* = duration from infection till infectiousness in mosquitoes (days)
*r* = recovery rate in humans (1/average duration of infectiousness in days)

The ratio of vectors to humans (*m*) is proportional to the basic reproduction number *R*_0_ (the number of secondary infections in humans each infectious human case would cause in a fully susceptible population). A higher *R*_0_ usually implies a higher incidence (our empirical outcome on which we have data), but the relationship between the two is rarely linear. *R*_0_ can be interpreted as the “epidemic potential” and therefore allows us to illustrate the potential role of *m* in dengue fever epidemics. Since *R*_0_ and incidence are not the same, we did not formally fit the model to the data. Incidence prediction would have required more complex dynamic transmission models, which were not necessary for our purposes.

On the basis of previous modeling work on dengue fever [27], we chose the following parameters for the estimation of *R*_0_: *a* = 1.0; β_mh_ and β_hm_ = 0.4; *p* = 0.8; *r* = 0.167. The Ross-MacDonald model implies that if *m* remains constant between areas of different human population density (vector and population numbers are proportional), then the resulting *R*_0_ will also be constant. Apart from this simple case we explored two scenarios: the first scenario assumed constant vector numbers independent of human numbers. We assumed this to reflect a situation where the lack of breeding sites severely limits mosquito numbers, and where mosquito numbers do not benefit from the availability of many human hosts for bloodfeeding (low potential for outbreaks).

In the second scenario, we assumed that the association between vector and host numbers initially increased but then plateaued, i.e., vectors benefit from increasing host numbers at low human population densities, but reach a plateau at higher host numbers. This scenario may be the most realistic, since mosquito numbers may be constrained at high human population densities, for example due to predators, lack of vegetation for feeding, or lack of breeding sites. We used the logistic function to represent this relationship, a function often used to simulate natural systems under limited resources.

For illustration, we chose parameters for the association between vectors and humans that resulted in an average of *R*_0_ = 1 (scenario 1, low potential for outbreaks) and *R*_0_ = 2 (scenario 2) across different human population densities. This choice was uncritical for the purposes of the model.

## Results

### Cohort Analysis

In the study population of around 350,000 residents living in 75,000 households, tap water and open wells were the most common types of water supply (each nearly 50%, Table 1). Between January 2005 and June 2008, 3,012 dengue fever cases required hospital admission during 1,219,025 person-years (PY) of follow up. Seventy-one percent of cases were clinically classified as dengue hemorrhagic fever. Dengue admission rate per 1,000 PY was highest in children between 5 and 15 y (Table 1). Adjusted admission rates decreased with distance to hospital and were lowest in households where no one had completed primary education. Admission rates were lowest in the highest wealth quintile (Table 1).

**Table 1:** Rate of dengue fever admission by socio-demographic and geographic characteristics.

*Characteristics*	*n* (%)	Crude Rate/1,000 PY	Adjusted Rate Ratio^a^	95% CI^a^
***Individual***				
**All**	349,994 (100)	2.6	—	2.5–2.7
**Age band (y)**				
≤2	9,295 (3)	3.9	1.0 (ref)	—
>2–5	21,952 (6)	3.8	0.96	0.73–1.27
>5–15	71,630 (20)	5.0	1.29	1.01–1.65
>15	247,108 (70)	1.8	0.47	0.37–0.60
**Gender**				
Male	172130 (49)	2.7	1.0 (ref)	—
Female	177,864 (51)	2.4	0.94	0.87–1.01
***Household***				
**All**	75,825 (100)	2.6	—	2.5–2.7
**Maximum level of education**				
Primary school not completed	4,960 (7)	1.4	1.0 (ref)	—
Primary school completed	21,532 (28)	2.5	1.67	1.30–2.13
Secondary school completed	25,853 (34)	2.7	1.76	1.38–2.25
High school completed	18,562 (24)	2.6	1.69	1.31–2.17
University completed	4,901 (6)	1.9	1.23	0.91–1.67
**Wealth level (quintiles)**				
1 (lowest)	20,435 (27)	2.4	1.0 (ref)	—
2	14,159 (19)	2.5	1.02	0.91–1.15
3	13,233 (17)	2.7	1.05	0.93–1.19
4	12,785 (17)	2.6	0.98	0.86–1.11
5 (highest)	15,165 (20)	2.3	0.83	0.73–0.94
**Distance to hospital (per km increase)**		—	0.94	0.93–0.96
**House composition**				
Brick/cement	68,030 (90)	2.5	1.0 (ref)	—
Mud brick	2,755 (4)	2.4	1.04	0.85–1.28
Wood/sticks	3,166 (4)	2.2	0.83	0.68–1.01
Other	1,842 (2)	1.9	0.78	0.58–1.05
**Population density (people residing within 100 m of HH)**				
0–50	9,681 (13)	2.5	1.0 (ref)	
51–100	13,540 (18)	2.9	1.09	0.94–1.26
101–200	16,493 (22)	3.2	1.14	0.99–1.31
201–400	12,373 (16)	2.2	0.75	0.64–0.88
401–800	15,139 (20)	1.9	0.61	0.52–0.72
801+	8,432 (11)	1.8	0.57	0.47–0.68
**Rural versus urban**				
Urban	33,821 (45)	2.2	1.0 (ref)	—
Rural	42,004 (55)	2.9	1.75	1.59–1.92
**Farming household**				
No	49,221 (65)	23	1.0 (ref)	
Yes	26,614 (35)	30	1.64	1.49–1.80
**Water supply**				
Tap water	35,491 (47)	2.1	1.0 (ref)	—
Bore hole/tube well	2,846 (4)	3.1	1.84	1.51–2.23
Open well	35,483 (47)	3.0	1.96	1.79–2.16
Rain water	564 (1)	1.2	0.78	0.43–1.41
River/pond/canal	971 (1)	2.0	1.82	1.23–2.68
Other	470 (1)	3.4	2.15	1.18–3.90

a All models included wealth, education, and distance to hospital.

HH, household ; ref, reference.

**Figure 2:**
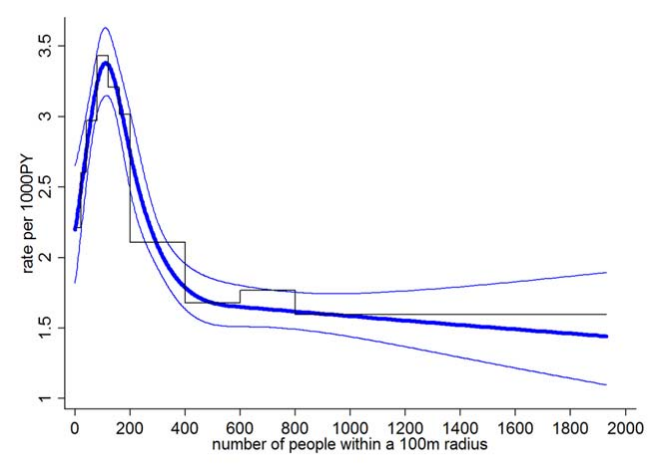
Dengue rate by number of people residing within 100 m. Staggered black line shows categorical analysis, smooth blue lines show the analysis with number of people as restricted cubic spline with 95% confidence bands (knots at 0, 100, 200, 300, and 600). All analyses adjusted for wealth, education, and distance to the nearest hospital.

Figure 2 shows a conspicuous peak in the (adjusted) rate of dengue fever at a relatively low population density of around 110 people residing within a 100-m radius of a study household. This figure corresponds to a population density of around 3,550 people/km^2^. In the study area, this population density is typical for rural villages, and some peri-urban areas.

In crude analysis, 61% of cases came from areas with a population density below 200 people within 100 m (6,360 people/km^2^), 75% from areas below 400 people within 100 m (12,730 people/km^2^).

Compared to the unadjusted model, adjusting for wealth, education, and distance to hospital increased the rate differences between moderate and high human population density, i.e., the peak rate of dengue fever at low-to-moderate population densities became more pronounced. Additional adjustment for age had little impact on the association between population density and dengue, since age was not associated with population density.

**Figure 3:**
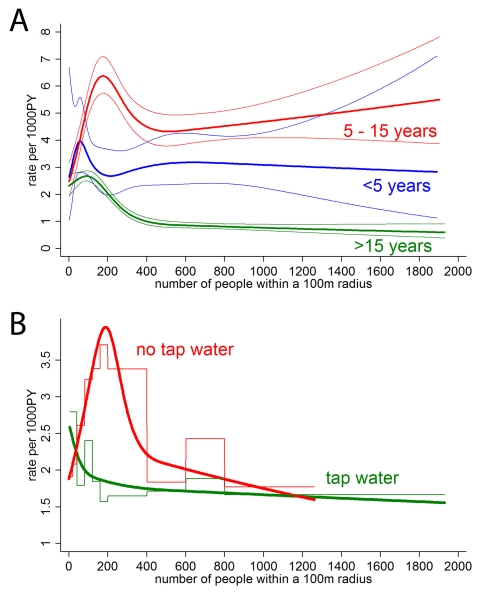
Subgroup analysis by age (A) and water supply (B). Staggered line (B only) shows categorical analysis, smooth line analysis with number of people as restricted cubic spline with 95% confidence bands (knots at 0, 100, 200, 300, and 600). All analyses adjusted for wealth, education, and distance to the nearest hospital.

On the basis of the adjusted model, we conducted subgroup analyses to identify potential effect modification (interaction), i.e., we explored whether the shape and position of the peak as displayed in Figure 2 depended on socio-demographic, geographic and clinical characteristics. We found that the location of the peak in the admission rate for dengue fever was at low-to-moderate human population densities for all age groups, but that the peak was somewhat less pronounced in children under 5 y (Figure 3A).

The peaks in the admission rate for dengue fever were similar in both epidemics, and between the more urban district of Nha Trang and the more rural district of Ninh Hoa. The position and the size of the peak did also not differ between classic dengue fever and dengue hemorrhagic fever.

We further stratified households into (1) being in a neighborhood (defined as a 100-m radius around each household) where more than 80% of households had access to tap water (named “tap water neighborhoods”); (2) those in neighborhoods where less than 20% of households had tap water (“well water neighborhoods”). Few neighborhoods fell in between these figures. Figure 3B shows that in well water neighborhoods largely lacking access to tap water, there is a distinct peak in dengue fever risk for households with around 190 people residing within 100 m (population density≈6,045 people/km^2^). In contrast, in tap water neighborhoods the highest risk was at very low human population densities.

Again adjusting for education, wealth, distance to hospital, and population density, we found that absence of tap water in an individual household increased the rate of dengue fever admission by a factor (rate ratio) of 1.66 (95% confidence interval [CI] 1.50–1.84). Additional adjustment for neighborhood tap water coverage (proportion modeled as cubic spline) reduced the rate ratio to 1.18 (95% CI 1.04–1.35), suggesting that neighborhood tap water supply largely (but not fully) explains the effect of water supply on dengue fever risk.

In Khanh Hoa Province, lack of water supply and a “critical” human population density were more common in rural than in urban areas. Areas defined as “rural” on the basis of local government information had a 1.75 higher rate of dengue fever (adjusted for education, wealth, distance to hospital) than “urban” areas (95% CI 1.59–1.92, Table 1). Additional adjustment for population density and tap water coverage (at household and neighborhood level) reduced the rate ratio to 1.11 (95% CI 0.96–1.27) suggesting that the rural/urban difference is largely due to these two factors.

### Scan Statistics

**Figure 4:**
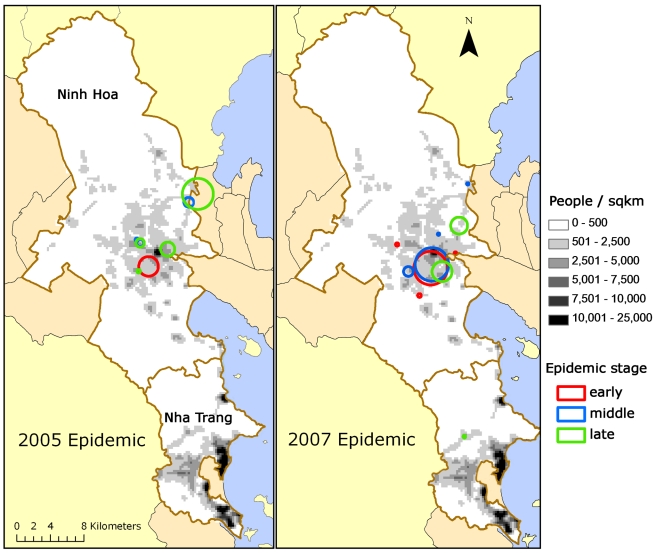
Clusters of dengue fever cases. (**A**) 2005 and (**B**) 2007 epidemics are shown by epidemic stage (early, middle, late).

Using an arbitrary cut-off of *p*<0.05, we identified 20 clusters (371 cases overall) with a mean population of 5,018 people (standard deviation [SD] 9,591) and 19 cases (SD 17). The mean of the cluster-level percentage of households without tap water was 86% (SD 8%, weighted by population size), i.e., the vast majority of households in dengue fever clusters lacked tap water. The mean number of residents within 100 m of a household at the cluster level was 172 (SD 48, weighted by population size), corresponding to a human population density of 5,473 people/km^2^ (see Table 2), which is similar to the population density with the highest risk identified through cohort analysis (Figure 3B). Figure 4 shows the location and geographic size of the clusters by epidemic stage, highlighting that densely populated areas were spared from major outbreaks.

**Table 2:** Characteristics of dengue fever clusters.

Year	Phase	*n* People in Cluster	*n* Cases	Mean Percent of Households without tap (SD)^a^	Mean *n* people (SD)^a^	*p*-Value
2005	Early	8,742	32	90 (30)	113 (49)	0.001
2005	Middle	824	32	97 (18)	60 (29)	0.001
2005	Middle	247	11	100 (0)	91 (38)	0.001
2005	Middle	1,920	9	100 (4)	131 (51)	0.014
2005	Late	2,624	26	99 (8)	54 (28)	0.001
2005	Late	2,383	23	100 (4)	131 (53)	0.001
2005	Late	6,000	31	85 (36)	143 (79)	0.001
2005	Late	466	7	100 (0)	107 (42)	0.001
2005	Late	178	4	98 (15)	173 (54)	0.015
2007	Early	29,220	75	85 (35)	179 (140)	0.001
2007	Early	507	7	100 (0)	135 (44)	0.001
2007	Early	132	5	100 (0)	56 (16)	0.001
2007	Early	263	7	99 (9)	82 (25)	0.003
2007	Middle	34,602	41	86 (35)	220 (169)	0.001
2007	Middle	93	4	100 (0)	55 (15)	0.001
2007	Middle	1,794	15	100 (0)	115 (32)	0.001
2007	Middle	279	6	28 (45)	165 (40)	0.005
2007	Late	1,264	10	46 (50)	64 (41)	0.001
2007	Late	8,043	18	75 (44)	137 (66)	0.001
2007/2008	Late	774	8	90 (30)	258 (68)	0.009

a Within a 100-m radius of each household.

### Mathematical Model

**Figure 5:**
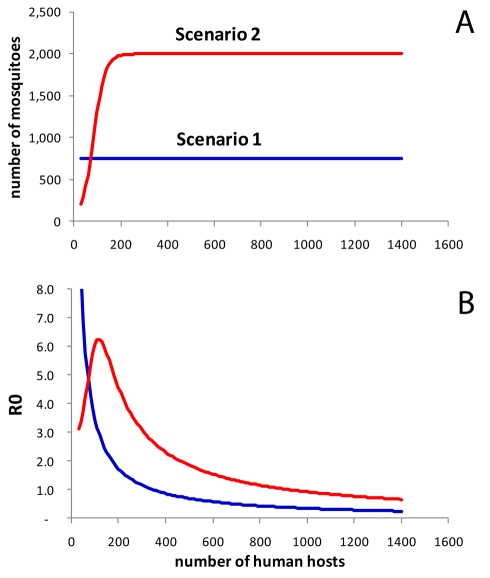
Simulation model. (A) Assumed associations between human population density (number of people in neighborhood) and number of mosquitoes. Scenario 1 assumes a constant number of mosquitoes (*N*_v_ = 750). The sigmoidal association (scenario 2, red) was specified as a logistic function *N*_v_ = *v*_max_/(*1*+*e* ^−k(h−I)^). In this example we used *v_max_* = 2,000 (maximum number of vectors), k = 0.04 (slope parameter), and I = 80 (inflection point). (B) Model results: *R*_0_ of dengue virus transmission by population density assuming constant vector numbers (scenario 1, blue), and a sigmoidal association (scenario 2, red).

In the first scenario (Figure 5A and 5B, blue), we assumed constant vector numbers independent of human population density, which resulted in a pattern not dissimilar to the risk of dengue in areas with good water infrastructure with the highest *R*_0_ (or incidence) occurring at very low human population densities (Figures 3B). We then assumed a sigmoidal association between host and vector numbers in the form of a logistic function (scenario 2, Figure 5A, red). This assumption produced an association between human population density and *R*_0_ with a conspicuous peak at low-to-moderate population densities, not dissimilar to the observed association between human population density and incidence (Figure 2). For illustration, we chose a turning point of the logistics function that resulted in a peak *R*_0_ at a similar position as in the real data; we found that a logistic function produced a distinct peak in *R*_0_ under most circumstances. Note that one could use many functions other than the logistic to represent the intended plateau effect in vector numbers (for example, a negative exponential function). We found that many functions starting at low vector numbers and leveling off at high human numbers produced a peak in *R*_0_ at intermediate human population densities.

Overall, the two scenarios provide an explanation for how provision of tap water fundamentally changes the epidemiology of dengue fever as a consequence of changes in vector numbers and vector ecology. Scarcity of breeding sites in the presence of tap water supply as the limiting factor for mosquitoes may result in vector numbers stabilizing at a low level, more or less independent of human population numbers (scenario 1). Thus, in scenario 1, and apparently also in the real data in areas with tap water supply, vector/host ratios compatible with intense dengue transmission may only occur at low human population densities.

## Discussion

We show that intense dengue virus transmission may occur in a remarkably narrow range of human population densities with a high mosquito/human host ratio in the absence of tap water supply. In our study area, the majority of cases were living in areas with low-to-moderate population density.

The findings may help to explain results from previous epidemiological studies. Dengue fever in Thailand has been shown to be more common in rural than in urban areas [14]. Barreto and colleagues found that dengue risk in Brazil was lower in vertical residential buildings than in more horizontally structured settlements [10]. Human population density in the latter may be more suitable for dengue transmission than in dense areas (in addition to potential differences in mosquito-breeding opportunities).

Our findings do not necessarily speak against urban centers contributing substantially to the spread of dengue [13]. The vector/host ratio in cities may be less suitable for intense transmission, but absolute case numbers can still be high. Dengue travels across regions in waves [13], and, as suggested by our results, is then amplified at places providing high vector/host ratios, for example, rural villages or low density areas with poor infrastructure within heterogeneous cityscapes [2]. Lack of a reliable water source in the immediate vicinity of a household requires constant planning and storing of water for convenience and in anticipation of shortages [28], providing breeding sites for *Aedes* mosquitoes [2],[3],[29]. Tap water provision appears to fundamentally change the ecology of dengue transmission (Figure 3), keeping vector numbers (as the model suggests) at a low level even if many hosts are available (Figures 3B and 5). Both the analysis and the model suggest that at generally low vector numbers (e.g., due to tap water supply), risk is highest at very low human population densities, since at higher population densities the few vectors predominantly feed on uninfected hosts. By assuming that at high human population densities the vector/host ratio is lower than at low-to-intermediate human population densities, our simple model offers a parsimonious explanation for the conspicuous peak in dengue risk at low human population densities, and the effect of tap water supply on vector abundance.

Dengue fever has a complex immunology not accounted for by our model, with antibodies against one serotype sometimes cross-protecting, sometimes enhancing disease severity following infection with a second serotype (antibody-dependent enhancement) [30]. The complex immunology of dengue virus infection is reflected by the cyclical occurrence of epidemics found in our study area (Figure 1) and many other settings. This pattern is most likely due to an interaction between the availability of susceptible hosts (e.g., children born after an epidemic), successive waves of different dengue virus strains, and climatic factors [30].

A study from Thailand suggests that transmission intensity may be positively related to mild or asymptomatic dengue but not severe dengue fever [31]. Conceivably, the peak in the risk of hospital admission for dengue at low-to-moderate human population densities may be due to (1) lower transmission intensity at high population densities or (2) higher immunity as a consequence of intense transmission at high population densities. We have no data on transmission intensity and cannot answer this question with certainty. In our view, the prominent role of lack of water supply (an assumed proxy for breeding sites) as a risk factor supports the view that hospitalizations are positively related to vector abundance and probably also transmission intensity. Also, the shape and position of the peak in dengue fever was similar between classic dengue fever and dengue hemorrhagic fever, which may indicate that population immunity did not greatly influence the position of the peak. If the low rate of hospital admissions at high human population densities were due to high immunity, one may expect this immunity to increase with age and the peak in dengue rate to move from higher to lower population densities with increasing age. We found no evidence for this (Figure 3A). Serological surveys in different age groups sampled in areas with different human population densities may in the future provide further clues.

In addition to the limitations of our model discussed above, our study is limited by methodological issues common to most large scale observational studies: bias, confounding, and imprecision. One source of bias may be due to potential differences in outmigration between population groups for which we had no data. Hospital admissions are biased towards more severe dengue underestimating the true disease burden [32], and towards more educated, wealthier groups living closer to the hospital, which may obscure a potential inverse association between wealth/education and rate of dengue. Confounding (e.g., due to socio-economic factors) does not seem a likely explanation for the findings. It may be difficult to think of a confounder associated with the exposure (human population density) and the outcome (dengue) that would be able to produce the conspicuous nonlinear association between population density and dengue, especially since adjusting for confounders tended to make the peak in dengue risk more pronounced.

Sensitivity and specificity of dengue rapid tests have been shown to vary depending on the setting and are subject to cross-reactivity, for example, due to malaria or leptospirosis [33], both of which are currently too rare in the study area to be of substantial impact.

Our human population density measure (people residing in a 100-m radius) is imprecise by not accounting for migration, travel, or death, and includes imprecision inherent to GPS data. Also, the site of infection may well differ from the site of residence. Further, we had no information on tap water reliability.

It could be important to understand why mosquito numbers appear to be constrained at high host densities despite ample opportunities for blood-feeding. If availability of breeding sites is the main limitation, breeding site reduction should then reduce dengue transmission. However, in areas with poor water infrastructure, dense human settlements may provide good breeding opportunities for *Ae. aegypti*, a mosquito using a wide range of artificial containers for laying eggs such as flower vases, toilet basins, water tanks, and jars [3]. If other factors (e.g., predators, lack of nutrition other than human blood) limit mosquito populations, reducing breeding sites may have little impact unless major efforts are made, such as the near-universal provision of tap water.

Ideally, all people should have access to reliable tap water, not only to reduce the burden of dengue [11], but also a range of other diseases associated with inadequate water supply such as diarrhea or trachoma, and to realize important economic benefits [28]. In many low-income settings, supplying everyone with tap water is not a realistic short-term goal. Our findings confirm, rather than contradict, the need for integrated approaches to reduce mosquito breeding around human settlements [5]–[7], but suggest that in the absence of tap water such efforts are an uphill struggle. Additional intervention measures in areas with a human population density critical for dengue virus transmission could increase the efficiency of vector control, especially since population density figures are relatively easy to obtain.

Our findings could apply to other viral infections transmitted by *Aedes* mosquitoes (e.g., Rift-Valley, West-Nile, Chikungunya, Yellow fever) and may be of relevance for other vector-borne infections, such as malaria or lymphatic filariasis. Vector biology and breeding behavior are likely to be major determinants of vector/host ratios and of whether an area is prone to outbreaks of a vector-borne disease.

## Abbreviations

CI: confidence interval
PY: person-years
SD: standard deviation
